# “Shining a LAMP” (Loop-Mediated Isothermal Amplification) on the Molecular Detection of Phytopathogens *Phytophthora* spp. and *Phytophthora cactorum* in Strawberry Fields

**DOI:** 10.3390/pathogens10111453

**Published:** 2021-11-10

**Authors:** Dominika G. Siegieda, Jacek Panek, Magdalena Frąc

**Affiliations:** Institute of Agrophysics, Polish Academy of Sciences, Doświadczalna 4, 20-290 Lublin, Poland; d.malarczyk@ipan.lublin.pl (D.G.S.); j.panek@ipan.lublin.pl (J.P.)

**Keywords:** Loop-Mediated Isothermal Amplification, *Phytophthora cactorum*, *Phytophthora* spp., rhizosphere, soil, shoots, roots, simple detection

## Abstract

Phytopathogenic microorganisms belonging to the genus *Phytophthora* have been recognized many times as causal agents of diseases that lower the yield of many plants important for agriculture. Meanwhile, *Phytophthora cactorum* causes crown rot and leather rot of berry fruits, mainly strawberries. However, widely-applied culture-based methods used for the detection of pathogens are time-consuming and often inaccurate. What is more, molecular techniques require costly equipment. Here we show a rapid and effective detection method for the aforementioned targets, deploying a simple molecular biology technique, Loop-Mediated Isothermal Amplification (LAMP). We optimized assays to amplify the translation elongation factor 1-α (EF1a) gene for two targets: *Phytophthora* spp. And *Phytophthora cactorum*. We optimized the LAMP on pure strains of the pathogens, isolated from organic plantations of strawberry, and successfully validated the assay on biological material from the environment including soil samples, rhizosphere, shoots and roots of strawberry, and with SYBR Green. Our results demonstrate that a simple and reliable molecular detection method, that requires only a thermoblock and simple DNA isolation kit, can be successfully applied to detect pathogens that are difficult to separate from the field. We anticipate our findings to be a starting point for developing easier and faster modifications of the isothermal detection methods and which can be applied directly in the plantation, in particular with the use of freeze-dried reagents and chemistry, allowing observation of the results with the naked eye.

## 1. Introduction

The reduction in harvest during the production of fruits, caused by pathogenic microorganisms and the diseases they bring to the plantation, is a severe obstacle in agriculture. *Phytophthora* species have been reported as causal agents for diseases in many crops and ornamental plants in the world [1,2,3,4,5,6]. The number of species recognized inside the genus and their hosts is constantly increasing [7,8], whereas *P. cactorum* has been reported as a soil-borne pathogen causing dieback mainly of the strawberry (*Fragaria × ananassa*) by both crown rot and leather rot of fruits [9]. The disease symptoms brought by *P.cactorum* are often misrecognized as those caused by different fungal pathogens. What is more, the pathogen has been recognized as being able to transmit not only on machine parts used in agriculture, but also in nursery seedlings and by water [10]. Finally, the *Phytophthora* spp. are not host-specific and can attack many plants, causing their dieback [1]. These four facts increase the severity of the infestation of fields with these pathogenic microorganisms. To implement appropriate preventive methods in the plantations, rapid and efficient detection of phytopathogens present in the fields is crucial. Assays deploying molecular biology techniques offer reliable and immediate detection in comparison to traditional identification methods based on the morphological attributes of the pure strains [11,12,13,14].

Loop-Mediated Isothermal Amplification (LAMP) is a highly effective isothermal DNA amplification technique, developed by Notomi’s team in 2000. The assay uses thermostable polymerase with a strand-displacement activity and two to three pairs of primers to amplify specific DNA fragments [15]. LAMP is characterized by four main advantages in comparison to other molecular techniques used for the detection of pathogens. First of all, the reaction is performed at a constant temperature; it does not require an expensive thermocycler and a water bath or thermoblock is sufficient to carry out the detection. Further, for the visualization of the results with the naked eye, fluorescent dye, such as SYBR Green I or hydroxy naphthol blue (HNB), can be added to the reaction mixture. Next, LAMP is 10–100 times more sensitive than Polymerase Chain Reaction [16] and requires lower amounts of input DNA. Finally, PCR inhibitors do not prevent detection with LAMP [17]. On the other hand, the main disadvantage of the technique is the fact, that the design of primers is complex, which restricts the development and optimization of the method only to specialists [18]. Nonetheless, this assay can be performed by non-specialists with ease. The usefulness of the reaction is also given by the fact that, with the use of a simple thermoblock and a fluorescent dye, the assay is suitable for use outside a well-equipped laboratory, including detection in the field conditions [19]. Therefore, new amplification technologies are useful for the development of rapid and field-deployable approaches to genetic diagnostics [20].

Loop-Mediated Isothermal Amplification has already been deployed in the detection of important strawberry pathogens with success [21,22,23,24,25,26,27,28]. Additionally, the LAMP assay for the detection of *Phytophthora* spp. on many plants, such as cucumber [29], soybean [30,31], potato [32,33,34,35,36], tomato [33], taro plants [37], lettuce [38], tobacco [39] and *Rhododendron* trees [16], has been reported. Although there are now numerous examples of LAMP applications in agriculture [40,41], animal health [42], and human medicine [43], there is still the need to develop new assays for the detection of the pathogenic organisms, especially dedicated to specific plants such as strawberry or for native strains of microorganism. Optimising specific detection assays developed for plantations occurring in a particular geographic location is very important, because of the possible genetic variation of site- or plantation-specific strains of pathogens. It is important to remember that organic plantations are characterized by the specific composition of the microbiome and minerals present in the soil and cooccurring in plant tissues which can inhibit the reaction, so it is important to develop detection methods for a given plant and pathogen population. Moreover, approaches that are more sensitive and combine rapid amplification with specificity are becoming an important diagnostic tool, especially for biological samples from the environment (shoots, roots, fruits, rhizosphere, soil) originating from organic cultivation, where chemical agents are not used. Moreover, LAMP for the detection of *Phytophthora* spp. nor *Phytophthora cactorum* strains found in strawberry fields has not been proposed so far.

Considering the aforementioned facts, this study aimed to develop and optimize an effective Loop-Mediated Isothermal Amplification method for the detection of the *Phytophthora* spp. and *Phytophthora cactorum* pathogens, which are a threat to strawberry plantations.

## 2. Results

### 2.1. Specificity of the Developed Reaction

**Figure 1:**
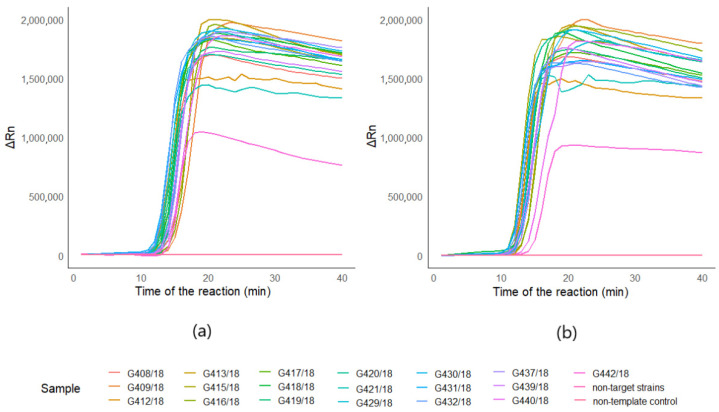
Amplification plots of LAMP detection. Amplification plots of 19 *Phytophthora* spp. (G408/18, G409/18, G412/18, G413/18, G415/18, G416/18, G417/18, G418/18, G419/18, G420/18, G421/18, G429/18, G430/18, G431/18, G432/18, G437/18, G439/18, G440/18, G442/18) pure strains and non-targeted DNA mixtures of *Botrytis* spp. (G276/18, G277/18, G321/18, G322/18, GG323/18), *Colletotrichum* spp. (G168/18, G170/18, G171/18, G172/18, G274/18 and *Verticillium* spp. (G294/18, G296/18, G297/18, G298, G299/18), deposited in the collection of LMEM; (**a**) amplification plots of the reaction for *Phytophthora cactorum* assay; (**b**) amplification plots of the reaction for *Phytophthora* spp. target.

**Figure 2:**
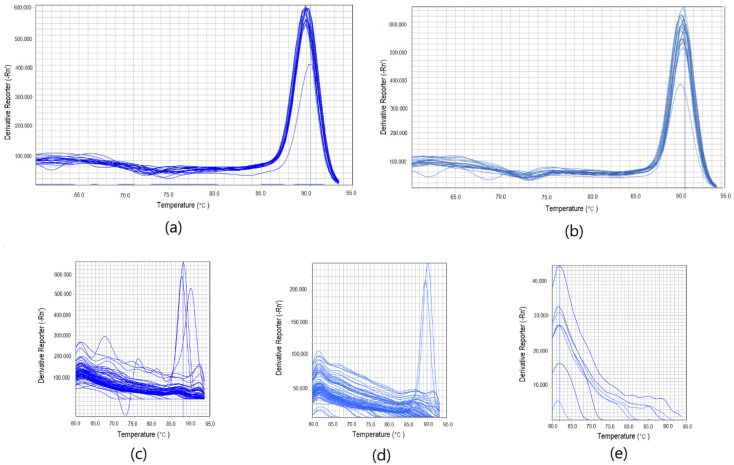
Melting curves of *Phytophthora* spp. (**a**) and *Phytophthora cactorum* (**b**) assays. Additionally, melting curves of chosen negative and positive reactions in biological samples from the environment for *Phytophthora* spp. (**c**) and *Phytophthora cactorum* (**d**) at peak signal at ~90 °C. The melting curve of non-targeted DNA samples including *Colletotrichum* spp., *Botrytis* spp. and *Verticillium* spp. (**e**) show the signal of the detection of primer-dimers at peak signal at ~63 °C.

Optimized assays for both of the targets: *Phytophthora* spp. and *Phytophthora cactorum* gave positive results for 19 strains (G408/18, G409/18, G412/18, G413/18, G415/18, G416/18, G417/18, G418/18, G419/18, G420/18, G421/18, G429/18, G430/18, G431/18, G432/18, G437/18, G439/18, G440/18, G442/18) (Figure 1a,b). Both of the assays did not amplify non-template controls, as well as non-targeted DNA (Figure 1a,b). The time of detection-Td (minute of the reaction when the maximum of the second derivative of normalized reporter value was reached) for positive reactions for *Phytophthora* spp. was 13.16, SD ± 0.83, and the Tm (melting temperature) was 89.92 °C, SD ± 0.18 (*n* = 19). For *P.cactorum*, the Td was 12.43, SD ± 0.85, and the Tm was 89.77 °C, SD ± 0.17 (*n* = 19) (T1, Supplementary Materials). Amplification plots for both of the targets are presented in Figure 1. Positive results for all of the tested *Phytophthora* spp. and *Phytophthora cactorum* strains suggest that the developed reaction is very specific. Melting curves of *Phytophthora* spp. and *Phytophthora cactorum* assays are pictured in the Figure 2a,b, with the peak of the signal at ~90 °C in positive reactions. Melting curves of chosen negative and positive reactions in biological samples from the environment for *Phytophthora* spp. (Figure 2c) and *Phytophthora cactorum* (Figure 2d) also showed peak signal at ~90 °C in positive reactions. The melting curve of non-targeted DNA samples (Figure 2e) shows the signal of primer-dimers at peak signal at ~63 °C.

### 2.2. Sensitivity of the Developed LAMP Assays

**Figure 3:**
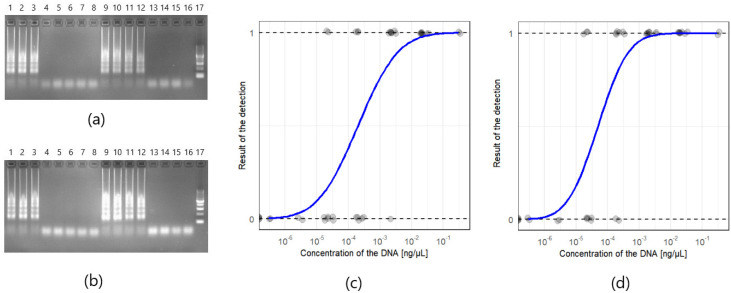
The detection limit of the LAMP detection method for *Phytophthora* spp. and *Phytophthora cactorum*: (**a**) Agarose gel of the detection limit of FastDNA Spin Kit for Feces kit (MP Biosystems) isolation method, for both targets *Phytophthora* spp. (wells 1–8) and *Phytophthora cactorum* (wells 9–16) of G408/18 strain. Order of the dilutions in each combination: 300 pg/µL (1 and 9), 30 pg/µL (2 and 10), 3 pg/µL (3 and 11), 300 fg/µL (4 and 12), 30 fg/µL (5 and 13) and 3 fg/µL (6 and 14), 300 ag/µL (7 and 15), NTC (non-template control, 8 and 16); (**b**) Agarose gel of the Plant & Fungi DNA Purification Kit (EURx) isolation method. Well 17 in (**a**) and (**b**) New England BioLabs 50 bp DNA Ladder. Characteristic for LAMP ladder-like patterns are visible on the lanes with positive reactions (1, 2, 3, 9, 10, 11, and 12); (**c**) Probit model of probability of the positive detection of *Phytophthora* spp. (*n* = 38) depending on the concentration of the DNA (isolation of the DNA was performed with the PrepMan Ultra and FastDNA Spin Kit for Feces kit and Plant & Fungi DNA Purification Kit); (**d**) Probit model for *Phytophthora cactorum* assay (*n* = 38).

Agarose gels for the detection limit of *Phytophthora* spp. and *Phytophthora cactorum* conducted on the DNA isolated from the G408/18 strain are presented in Figure 3a,b. The detection of the *Phytophthora* spp. target was achieved in the samples with the concentration range from 0.3 ng/µL to 3 pg/µL for both of the tested isolation methods. *Phytophthora cactorum* assay was more sensitive, reaching the detection of 300 fg/µL for both of the isolation methods. What is more, no amplification was observed in non-template controls. The results suggest that the detection limit for the optimized reaction is 3 pg/µL for *Phytophthora* spp. and 300 fg/µL for *Phytophthora cactorum*, regardless of the tested DNA isolation method. Probit models of the positive reaction of the detection of *Phytophthora* spp. (*n* = 38) and *Phytophthora cactorum* (*n* = 38) are shown in Figure 3c,d, respectively.

### 2.3. Colorimetric Validation

**Figure 4:**
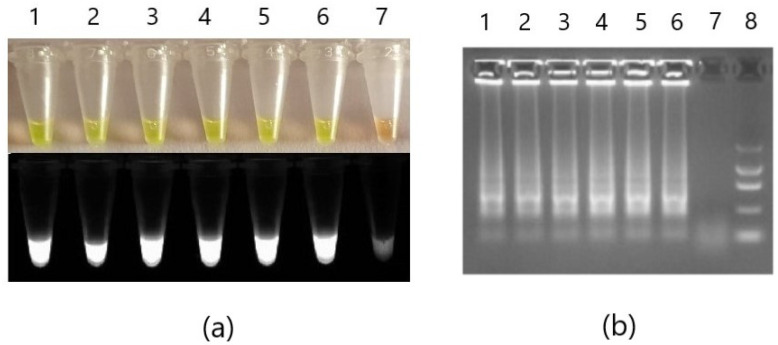
Colorimetric validation of the developed detection methods. LAMP detection for *Phytophthora cactorum* of strains G415/18 (1 and 4), G416/18 (2 and 5), and G417/18 (3 and 6) conducted in thermo-block in 65 °C for 30 min. After the reaction, SYBR Green I (1:9) dye was added to the mixtures for visualization of the results in visible light and UV (**a**). Samples were then electrophoresed in 2% agarose gel (**b**). Positive reactions samples 1-6; non-template control sample 7. Additionally, in (**b**), the A&A Marker I was added in well 8.

After the detection of *Phytophthora cactorum* performed on strains G415/18, G416/18, and G417/18 (each in duplicate) in the thermoblock, 1.5 µL of SYBR Green I dye was added into each reaction tube with sterile pipette tips. Reaction mixtures immediately changed color from transparent to yellow in positive samples and into orange in the negative control (Figure 4a). After the examination under the UV light, the positive samples showed bright fluorescence, whereas negative samples were very low. An attempt at visualization of the reaction products in 2% agarose gel revealed ladders in positive reactions, which confirms amplification of LAMP products (Figure 4b).

### 2.4. Biological Samples from the Environment

**Figure 5:**
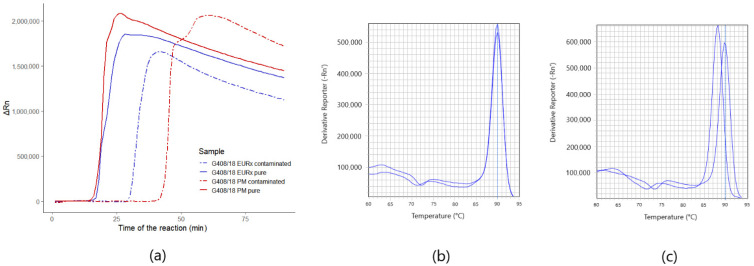
Amplification plots and melting curves of *Phytophthora* spp. assay: (**a**) amplification plots for the detection of *Phytophthora* spp. assay of pure G408/18 strain and contaminated biological material from the environment with the DNA of G408/18, isolated with FastDNA Spin Kit for Feces kit (MP) and Plant & Fungi DNA Purification Kit (EURx); (**b**) melt curves of pure and contaminated samples isolated with FastDNA Spin Kit for Feces kit (MP Biomedicals); (**c**) melt curves of pure and contaminated material samples isolated with Plant & Fungi DNA Purification Kit (EURx).

In both of the DNA samples of strain G408/18, detected with the *Phytophthora* spp. assay and isolated with FastDNA Spin Kit for Feces kit (MP Biomedicals) or Plant & Fungi DNA Purification Kit (EURx), the detection time of the environmental sample contaminated with the DNA of G408/18 pure strain environmental sample lengthened in comparison to pure samples of G408/18. Namely, the time of the detection in Plant & Fungi DNA Purification Kit (EURx) sample lengthened from the 17th to 30th minute and FastDNA Spin Kit for Feces kit (MP Biomedicals) from the 18th to 43rd (Figure 5a). What is more, the detection time of contaminated diluted reactions lengthened when compared to the reactions diluted in DirectQ water. Additionally, for the sample isolated with FastDNA Spin Kit for Feces kit (MP Biomedicals), Tm of contaminated diluted reaction decreased from 89.95 °C to 87.78 °C (Figure 5c). In the sample isolated with the Plant & Fungi DNA Purification Kit (EURx) kit, no change of melting temperature was noted (Figure 5b). The detection of the targets in biological samples from the environment was extended to 90 min, due to the fact, that the inhibitors from the environment that co-isolate with the DNA extend the time required for the detection.

In the detection of *Phytophthora* spp. in biological samples from the environment, 4 out of 348 samples derived in organic plantations of strawberries located in Eastern Poland gave positive results (1%). All of the samples with positive detection came from four different plantations, where the symptoms of the disease have not been recognized in the year of the sampling. The 478/19 bulk soil sample on Aprica plantation from the field 13 and the detection started after 65th minute, and the Tm was 90.15 °C. Sample 490/19 was also a bulk soil sample of Aprica cultivar from field 14. Detection started after the 35th minute and the Tm was 87.77 °C. The sample 48/19C was a bulk soil sample from fields 1 and 2. The detection started after the 66th minute with the Tm of 88.18 °C. Finally, positive sample 1632/20 was a fruit of Rumba cultivar, plantation 15, where the detection started after 66th minute and Tm was 90.32 °C (Table S1).

The *Phytophthora cactorum* assay gave positive results in 13 biological samples from the environment (4%). Sample 385K/19 was a root of an Aprica cultivar, plantation 4. The detection started from the 23rd minute, Tm was 91.09 °C; next, 449/19K for the root sample of Dipred from the plantation 10, where the previous year the symptoms of the disease caused by *Verticillium* spp. and *Phytophthora* spp. were identified. Detection time was 32 min and Tm was 90.13 °C. Sample 45/19C was bulk soil of Aprica cultivar, plantation 11. The remaining positive results were noted in the same field 15, and all of the samples were strawberry fruit samples. The time of detection ranged from 38 to 86 min and the Tm from 88.22 to 89.33 °C. (Table S1).

The peak of melting curves for the biological samples from the environment where the *Phytophthora* spp. gave positive results ranged from 87.77 to 90.32 °C, whereas for pure strains the range was 89.68–90.05 °C. For the *Phytophthora cactorum* assay performed on biological samples from the environment, the range of Tm was between 87.66 and 91.09 °C, whereas for pure samples this was within 89.5–90.05 °C.

## 3. Discussion and Conclusions

**Figure 6:**
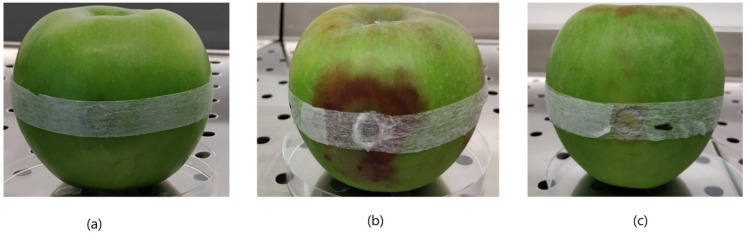
Apple trap method for baiting *Phytophthora* spp. pathogens. First, fragments of infected strawberry tissues were placed inside surface-sterilized apple fruit (**a**) and incubated at 22 °C for 10 d in a plastic bag. Infected apple tissues (**b**) changed color from green to brown, and softened. Negative control (**c**) remained unchanged.

Rapid and efficient identification of the pathogens present in a given field is very important as it allows the implementation of proper protection methods and significantly reduces losses related to the spread of the disease caused by microorganisms. Common, traditional plate-culture-based methods, as well as the apple trap method described previously [44] and in this work (Figure 6) for the isolation of pure strains of microorganism from the environment, are characterized with many disadvantages. These methods require a long incubation time and are inconvenient for many samples tested at the same time, when it is necessary to quickly diagnose the disease and the quality of the plantation, taking into account soil, plant, and fruit. Traditional identification methods based on the observation of microstructures of pathogens do not offer sufficient certainty when it comes to valid identification, as opposed to molecular techniques [45]. On the contrary, molecular methods of identification allow detection of the contamination in the field with pathogens before the manifestation of the disease in plants. The presence or absence of a particular pathogen in the field can give a clear indication of whether to start a new plantation. What is more, the results of the molecular detection of the pathogen also give a clear answer whether undertaken agrotechnical measures aimed at the removal of pathogens were effective. However, it is worth mentioning that, in some cases, it might be worth using trap methods and then to perform LAMP detection of these phytopathogens.

For the molecular detection of the *Phytophthora* spp. with the Polymerase Chain Reaction (PCR), different markers were used, such as ITS1, ITS2 of ribosomal RNA [46,47,48], or cytochrome oxidase I gene (COX1) [49]. Nonetheless, the real-time PCR method was also optimized for the detection of this pathogen. ITS markers were deployed in the detection of *Phytophthora* spp. in strawberry plantations [50,51]. Enolase (ENOL), ras-like protein (YPT1), and HSP90 genes were also targeted for this aim [52]. Finally, Loop-Mediated Isothermal Amplification is a relatively new detection method, adopting molecular biology, and has been deployed many times in the detection of plant pathogenic fungi and oomycetes on various plants of agricultural significance [41,53,54,55,56,57]. The method has been reported as an efficient tool for the detection of strawberry pathogens, as in [23,24,25,26,27]. In 2017, Khan’s team compared the detection of *Phytophthora infestans* with PCR, nested PCR, real-time PCR, and LAMP with the application of primers for the YPT1 gene. The team concluded that the LAMP was the most sensitive assay out of the tested methods, being 10 times more sensitive than nested PCR and 100 times more sensitive compared to real-time PCR [58].

LAMP is characterized by several advantages, such as high sensitivity of the reaction, high specificity, and constant thermal conditions of the assay. Among them, the fact that the assay has the potential to be used in field conditions seems to be the most important in sustainable phytopathogen control. Due to the fact that the reaction does not require thermal cycling, as opposed to PCR or qPCR, the water bath or a thermoblock is sufficient to provide constant temperature in order to perform the analysis. As we tested 3 DNA isolation kits in the current study (FastDNA Spin Kit for Feces kit, MP Biomedicals, Plant & Fungi DNA Purification Kit, EURx, and PrepMan Ultra Sample Preparation Reagent—Applied Biosystems by Thermo Fisher Scientific), we proved that the LAMP is not dependent on a specific DNA isolation method. Additionally, as reported in the past, direct evaluation of the results was performed with the addition of chemistry such as calcine [59], hydroxy naphthol blue (HNB) dye [60], or SYBR Green [54,61] as in this study, allowing observation of the change in the color of the positive samples by the naked eye or in ultraviolet light (UV). What is more, lyophilized forms of LAMP reagents [62] can be taken into consideration when talking about the in-field application of the method. Those facts suggest that the assay has a wide range of adaptation possibilities for current conditions in a given laboratory and outside the laboratory. The simplicity of the method application could lead to simple field-deployable products in the future, allowing for rapid detection of plant pathogens from the biological samples from the environment, without costly equipment and highly specialized laboratory staff.

In conclusion, the LAMP assay using primer sets developed in this study successfully detected *Phytophthora* spp. and *Phytophthora cactorum* isolates acquired from organic plantations of strawberry. Moreover, the LAMP assay using developed primers and optimized conditions detected these pathogens rapidly and simply in biological samples from the environment, collected from strawberry plantations. Therefore, the results demonstrated that the LAMP assay with developed primer sets can be used for routine detection and monitoring of strawberry plantations for the presence of *Phytophthora* spp. and *Phytophthora cactorum*.

## 4. Materials and Methods

### 4.1. Obtaining Pure Cultures of Phytophthora spp.

The phytopathogenic organisms used in the development of this assay were gained from organic plantations of strawberries located in Eastern Poland. Infected plant tissues were placed on Petri dishes with Carrot Agar (CA) or Potato Dextrose Agar (PDA) media and incubated at 22 °C until cultures appeared on the plates. Then, they were further subcultured onto new CA or PDA media until pure cultures were obtained [63]. As this method was not efficient enough for some of the strains, the apple trap method was also deployed (Figure 6), as reported in guidelines of the Main Inspectorate of Plant Health and Seed Inspection in Poland [44], to increase the effectiveness of *Phytophthora* spp. isolation. Granny Smith green apples were washed with detergent water, rinsed with distilled water, and 70% ethanol, and, after such sterilization, fragments of strawberry roots identified visually as infected were placed in the slot in the apple fruit made with a sterile cork borer. The strawberry tissue was covered with cut-out apple tissue and then sealed with a porous adhesive tape (3M Micropore). An apple had three slots for infested roots, and negative control was also made on each trap with no diseased strawberry tissue inside. An apple trap was then placed in a plastic bag and incubated at 22 °C for 10 d [44]. Thereafter, infected apple tissues were placed on the PDA and further subcultured until pure cultures developed on the medium.

### 4.2. Isolation of the DNA from Pure Strains and Biological Samples from the Environment

For identification purposes as well as to determine the specificity of the reaction and the detection limit assay, the DNA of *Phytophthora* spp., *Botrytis* spp., *Colletotrichum* spp., and *Verticillium* spp. was isolated with PrepMan Ultra Sample Preparation Reagent (Applied Biosystems by Thermo Fisher Scientific, Waltham, MA, USA), following the manufacturer’s protocol. Then, the DNA samples were diluted 100 times in DirectQ water before the molecular analysis. Following, the D2 large subunit region of the fungal rDNA was amplified and sequenced as described by Pertile et al. [64] with a modified purification step, using Clean DTR (CleanNA, Qaddinxveen, Netherlands). The information regarding pure strains of *Phytophthora* spp., *Botrytis* spp., *Colletotrichum* spp., and *Verticillium* spp. used in this study is gathered in Table 1.

Isolation of the genomic DNA from pure strains of the *Phytophthora* sp. (G408/18) for the detection limit assays was performed with FastDNA Spin Kit for Feces kit (MP Biomedicals, Solon, OH, USA) and Plant & Fungi DNA Purification Kit (EURx, Gdańsk, Poland). Before the isolation, pure strains of the pathogen were grown at 22 °C for 10 days in 15 mL conical flasks in Potato Dextrose Broth (PDB). After the incubation, the liquid cultures were centrifuged for 15 min in 4500× *g*, the supernatant was discarded and cultures were washed with 5mL sterile water three times. In the meantime, for the Plant & Fungi DNA Purification Kit (EURx), homogenization tubes were prepared as described by Panek and Frąc [65]: 2 mL cork-cap tubes were filled with 0.5 g of 3.15 mm diameter and 0.25 g of 1.4 mm diameter glass beads and sterilized. Then, the mycelium was sterilely transferred into the prepared tubes and homogenized with Fast-Prep instrument (MP Biomedicals) at 4 m/s for 10 s and the DNA was isolated according to the manufacturer’s protocol. Obtained DNA was eluted with 100 µL of Tris-HCl buffer (10 mM Tris-HCl, pH 8.5) and stored at −22 °C until used.

For the DNA isolation with the FastDNA Spin Kit for Feces, washed mycelia were first placed into the 2 mL tubes with the 0.1 mm silica spheres, 1.4 mm ceramic spheres, 4 mm glass ball, 825 µL phosphate buffer, and 275 µL PLS reagent. The samples were then centrifuged for 5 min in 14,000× *g* and supernatant was discarded. Homogenization was conducted with FastPrep 24 instrument (20 s, 6 m/s) with the 978 µL of sodium phosphate buffer and 122 µL of MT buffer. After the centrifugation (15 min, 14,000× *g*), the supernatant was transferred into the new tube with 250 µL of PPS buffer, mixed, and incubated (10 min, 4 °C). After another centrifugation (2 min, 14,000× *g*), the supernatant was transferred into a 5 mL tube with 1 mL of Binding Matrix Solution and mixed on a rotator for 5 min. The samples were then centrifuged (2 min, 14,000× *g*), the supernatant was discarded and the pellet was washed with 1 mL of Wash Buffer 1 and transferred into SPIN Filter columns. The samples were then centrifuged for 1 min in 14,000× *g* and the filtrate was discarded twice. The second wash was performed similarly, with 500 µL of Wash Buffer 2 and 2 min centrifuge run (14,000× *g*). Finally, 100 µL of the Elution buffer (TES) was pipetted onto the filter and centrifuged for 2 min (14,000× *g*). The obtained filtrate was then 10-fold diluted in nuclease-free deionized water and stored at −22 °C until used. The quality and quantity of the genetic material isolated with both of the methods were verified by electrophoresis and with Nanodrop 2000 instrument (ThermoFisher Scientific, Waltham, MA, USA).

The DNA extraction from biological samples from the environment was conducted with the FastDNA Spin Kit for Feces kit, using 0.5 g of soil or 0.25 g of strawberry fruit tissue and according to the manufacture’s protocol with modifications described earlier. Additionally, the homogenization was lengthened to 40 s. The filtrate obtained after the elution was 10-fold diluted in nuclease-free deionized water and stored at −22 °C until performing a detection.

### 4.3. Primers Development and LAMP Optimization

**Figure 7:**
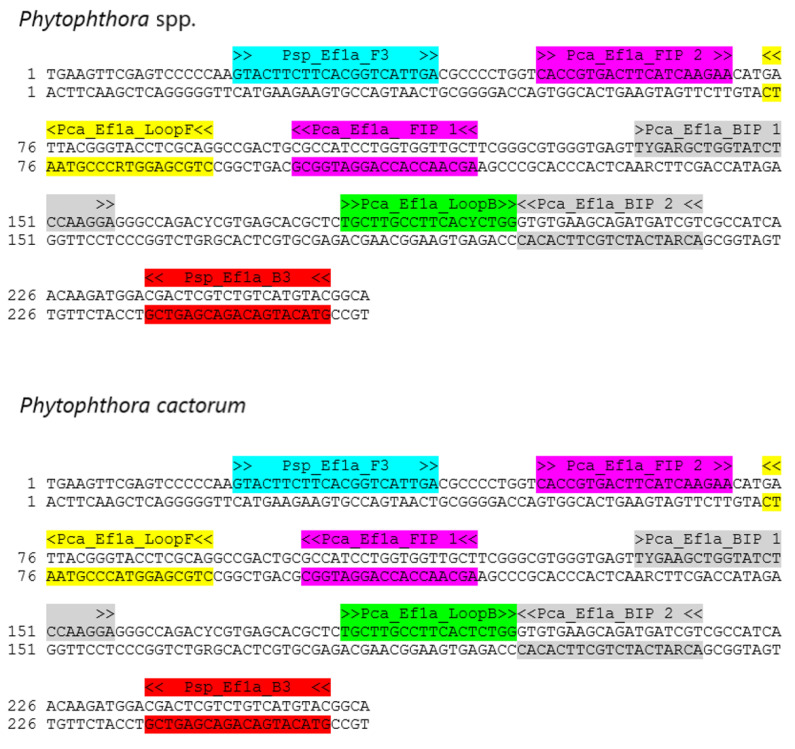
The localization of primers in the fragment of EF1α gene chosen for detection of *Phytophthora* spp. and *Phytophthora cactorum* on contig of sequences of *Phytophthora* spp. strains used for the design of primers.

For the LAMP assay development, the translation elongation factor 1-alpha (EF1α) gene was chosen as a genetic marker after the GenBank database [66] review. Gene was sequenced as described by Frąc et al. [67]. Then, obtained sequences of EF1α fragments of 19 *Phytophthora* spp. strains collected from strawberry plantations were deposited in GenBank (Table 1) and aligned in MEGA software [68] with several DNA fragments of different representatives from the genus, retrieved from the GenBank database [66]. Further, the possible LAMP primers were designed with the LAMP Designer v.1.13 software (OptiGene Limited, Horsham, UK) and validated in silico with BLAST [69]. All of the oligonucleotides were synthesized in Genomed S.A. (Warsaw, Poland). The Psp_Ef1a_F3 and Psp_Ef1a_B3 primers were used as outer primer pairs for both targets—*Phytophthora* spp. and *Phytophthora cactorum* (Patent applications P.437111 and P.437110, respectively). The information regarding sequences of the primers is gathered in Table 2 and the location of the primers in the contig of EF1α gene fragment is presented in Figure 7.

LAMP assays for the method optimization were performed in the 7500 Fast thermocyclers (Applied Biosystems, Foster City, CA, USA) and the total reaction volume was 10 µL. The mixture consisted of 6 µL of Isothermal MasterMix (ISO-001, OptiGene, Horsham, UK), 3 µL of primer mix (Table 2), and 1 µL of the DNA sample. The Isothermal MasterMix consisted of fluorescent dye detected by the FAM channel and an isothermal GspSSD polymerase with strand-displacement activity [24]. The reactions were conducted at 65 °C for 40 min with the reading of the fluorescent signal after every minute. After every reaction, the melting curve analysis was performed (65 °C to 95 °C, ∆0.016 °C/s).

To determine the specificity of the reaction, both primer sets were tested on 19 *Phytophthora* spp. strains (G408/18, G409/18, G412/18, G413/18, G415/18, G416/18, G417/18, G418/18, G419/18, G420/18, G421/18, G429/18, G430/18, G431/18, G432/18, G437/18, G439/18, G440/18 and G442/18) isolated from organic plantations of strawberry and deposited in the Laboratory of Molecular and Environmental Microbiology (LMEM) collection (Table 1). Additionally, the reaction was carried out with the mix of the non-targeted DNA isolated from five strains of *Botrytis* spp. (G276/18, G277/18, G321/18, G322/18 and GG323/18), five strains of *Colletotrichum* spp. (G168/18, G170/18, G171/18, G172/18 and G274/18), and five strains of *Verticillium* spp. (G294/18, G296/18, G297/18, G298, and G299/18). Each of the representatives of non-targeted species was combined in an equal amount into one Eppendorf tube, then 1 µL of the non-targeted DNA mixture was added into reaction and run in identical conditions as targeted samples in biological triplicates.

### 4.4. Detection Limit

To establish the detection limit for the optimized reactions, serial 10-fold dilutions of the DNA isolated with three different isolation kits were prepared. Pure strain G408/18 isolated with FastDNA Spin Kit for Feces kit and Plant & Fungi DNA Purification Kit DNA concentrations of 300 pg/µL, 30 pg/µL, 3 pg/µL, 300 fg/µL, 30 fg/µL, and 3 fg/µL were added into the reaction mixtures for *Phytophthora* spp. and *Phytophthora cactorum* assays and fluorescent signal was measured during reactions. Additionally, electrophoresis in the agarose gel (2%, 6 V/cm, 40 min) with Color Load (10 x, EURx, Gdańsk, Poland) and the Marker I (A&A Biotechnology, Gdynia, Poland) was conducted for the initial check of results (Figure 4). Further, serial 10-fold dilutions of the G415/18, G416/18, and G417/18 strains isolated with PrepMan Ultra were made with sterilized DirectQ water. Starting concentration of the DNA dilutions was 20 pg/µL and the lowest 20 fg/µL. Probit model of the positive result of the detection of *Phytophthora* spp. and *Phytophthora cactorum* was calculated using RStudio v.1.4.1103 with 43 observations for each assay.

### 4.5. Colorimetric Approach

To ensure the usefulness of the developed method for the detection of *Phytophthora* spp. and *Phytophthora cactorum* pathogens outside well-equipped conditions, reaction with undiluted strains G415/18, G416/18, and G417/18 was carried out in the thermo-block (ThermoStat Plus, Eppendorf AG, Hamburg, Germany) at 65 °C for 30 min. To improve the visualization of the results with the naked eye, the reaction was conducted in 20 µL and 1.5 µL of 10 times diluted SYBR Green I (ThermoFisher Scientific, Waltham, MA, USA) was added after the reaction, as the dye inhibits the reaction. Reaction results were also visualized on an agarose gel (2%, 6 V/cm, 40 min) and in UV light (Figure 4).

### 4.6. Validation of the Assay in Biological Samples from the Environment

As it is known, contaminants derived from biological samples from the environment may co-isolate during the DNA extraction. The reaction verifying how the contaminants of the DNA samples isolated from the environment may affect the effectiveness of the reaction was performed. For this purpose, two samples of the DNA, isolated with MP Biomedicals kit and EURx Plant and Fungi isolation kit from a pure sample of G408/18 were added into the environmental sample (recognized as not contaminated with *Phytophthora* spp. beforehand) in 1:9 proportion. Additionally, pure samples of the G408/18 were as well diluted 10-fold in sterile water. Then, the detection of *Phytophthora* spp. was performed on four types of samples: pure strain isolated with EURx, a pure strain isolated with MP, environmental sample contaminated with the G408/18 isolated with EURx, and environmental sample contaminated with the G408/18 isolated with MP. The results obtained during this step were then employed to decide if it is reasonable to increase the length of the environmental assay due to loss of the reaction sensitivity.

For validation of the usefulness of the developed detection method and its potential applicability, the assay was performed on 348 various biological samples from the environment derived from organic plantations of strawberries in July 2019 and 2020. The samples collected in 2019 were divided into categories, according to the plantation (14 different plantations), cultivars of strawberry: (Honey, Aprica, and Dipred), and type of the collected sample (rhizosphere, bulk soil, strawberry roots, and shoots). Samples collected in 2020 were all samples of strawberry fruits. The information regarding collected biological samples from the environment and positive results are gathered in Table S2.

## Figures and Tables

**Table 1:** Fungal strains used for the LAMP assay development.

Fungal Genus	Isolate Code LMEM	Isolation Source	Method of Obtaining Pure Strain	The Accession Number of D2LSU Sequences in GenBank	The Accession Number of EF1α Sequences in GenBank
*Phytophthora* spp.	G408/18 *	Strawberry plants, IA PAS	CA ^a^	MT126670.1	MW715837
	G409/18 *	Strawberry plants, IA PAS	CA ^a^	MT126671.1	MW715838
	G412/18 *	Strawberry roots, IA PAS	apple trap	MT126672.1	MW715839
	G413/18 *	Strawberry roots, IA PAS	apple trap	MT126673.1	MW715840
	G415/18 *	Strawberry roots, IA PAS	apple trap	MT126674.1	MW715841
	G416/18 *	Strawberry roots, IA PAS	apple trap	MT126675.1	MW715842
	G417/18 *	Strawberry roots, IA PAS	apple trap	MT126676.1	MW715843
	G418/18 *	Strawberry roots, IA PAS	apple trap	MT126677.1	MW715844
	G419/18 *	Strawberry plants, IA PAS	PDA ^b^	MT126678.1	MW715845
	G420/18 *	Strawberry plants, IA PAS	PDA ^b^	MT126679.1	MW715846
	G421/18 *	Strawberry plants, IA PAS	CA ^a^	MT126680.1	MW715847
	G429/18 *	Strawberry roots, IA PAS	apple trap	MT126681.1	MW715848
	G430/18 *	Strawberry roots, IA PAS	apple trap	MT126682.1	MW715849
	G431/18 *	Strawberry plants, IA PAS	PDA ^b^	MT126683.1	MW715850
	G432/18 *	Strawberry plants, IA PAS	PDA ^b^	MT126684.1	MW715851
	G437/18 *	Strawberry roots, IA PAS	apple trap	MT126686.1	MW715852
	G439/18 *	Strawberry roots, IA PAS	apple trap	MT126687.1	MW715853
	G440/18 *	Strawberry roots, IA PAS	apple trap	MT126688.1	MW715854
	G442/18 *	Strawberry roots, IA PAS	apple trap	MT126690.1	MW715855
*Colletotrichum* spp.	G168/18	Strawberry fruits, IA PAS	PDA ^b^	MT126804.1	-
	G170/18	Strawberry fruits, IA PAS	PDA ^b^	MT126805.1	-
	G171/18	Strawberry fruits, IA PAS	PDA ^b^	MT126802.1	-
	G172/18	Strawberry fruits, IA PAS	PDA ^b^	MT126803.1	-
	G274/18	Strawberry fruits, IA PAS	PDA ^b^	MT126807.1	-
*Botrytis* spp.	G276/18	Strawberry roots, IA PAS	PDA ^b^	MT154303.1	-
	G277/18	Strawberry roots, IA PAS	PDA ^b^	MT154304.1	-
	G321/18	Strawberry roots, IA PAS	PDA ^b^	MT154305.1	-
	G322/18	Strawberry roots, IA PAS	PDA ^b^	MT154306.1	-
	G323/18	Strawberry roots, IA PAS	PDA ^b^	MT154307.1	-
*Verticillium* spp.	G294/18	Strawberry roots, IA PAS	PDA ^b^	MT133317.1	-
	G296/18	Strawberry roots, IA PAS	PDA ^b^	MT133320.1	-
	G297/18	Strawberry roots, IA PAS	PDA ^b^	MT133316.1	-
	G298/18	Strawberry roots, IA PAS	PDA ^b^	MT133318.1	-
	G299/18	Strawberry roots, IA PAS	PDA ^b^	MT133319.1	-

* Fungal strains used for the in silico design of the primers; IA PAS: Institute of Agrophysics, Polish Academy of Sciences; LMEM: Laboratory of Molecular and Environmental Microbiology, IA PAS; ^a^: strain obtained with culturing of plant roots on Carrot Agar; ^b^: strain obtained with culturing of plant roots on Potato Dextrose Agar.

**Table 2:** Sequences of the primers designed for the LAMP assays for the detection of *Phytophthora* spp. and *Phytophthora cactorum* (Patent applications P.437111 and P.437110, respectively).

Marker	Target	Primer Name *	The Sequence of the Primer 5′–3′	Concentration
translation elongation factor 1-α (EF1a) gene	*Phytophthora* spp.	Psp_Ef1a_F3	GTACTTCTTCACGGTCATTGA	0.2 µM
		Psp_Ef1a_B3	GTACATGACAGACGAGTCG	
		Psp_Ef1a_FIP	AGCAACCACCAGrATGGC|CACCGTGACTTCATCAAGAA	0.8 µM
		Psp_Ef1a_BIP	TyGArGCTGGTATCTCCAAGGA|ACrATCATCTGCTTCACAC	
		Psp_Ef1a_LoopF	CTGCGAGGTrCCCGTAATC	0.4 µM
		Psp_Ef1a_LoopB	TGCTTGCCTTCACyCTGG	
	*Phytophthora cactorum*	Pca_Ef1a_FIP	AGCAACCACCAGGATGGC|CACGTGACTTCATCAAGAA	0.8 µM
		Pca_Ef1a_BIP	TyGAAGCTGGTATCTCCAAGGA|ACrATCATCTGCTTCACAC	
		Pca_Ef1a_LoopF	CTGCGAGGTACCCGTAATC	0.4 µM
		Pca_Ef1a_LoopB	TGCTTGCCTTCACTCTGG	

* Both sets of primers have the same F3 and B3 primer pair.

## Data Availability

Not applicable.

## Supplementary Materials

The following are available online at https://www.mdpi.com/article/10.3390/pathogens10111453/s1, Table S1: The time of detection-Td (minute of the reaction when the maximum of 2nd derivative of normalized reporter value was reached), and Tm (melting temperature) for positive reactions for *Phytophthora* spp. and *Phytophthora cactorum*; Table S2: Results of the detection of *Phytophthora* spp. and *Phytophthora cactorum* in environmental samples of organic strawberry fields. samples collected in 2019 and 2020.
