# Supplementary material for: “Shining a LAMP” (Loop-Mediated Isothermal Amplification) on the Molecular Detection of Phytopathogens Phytophthora spp. and Phytophthora cactorum in Strawberry Fields
Source: Pathogens. 2021 Nov 10;10(11):1453. doi: 10.3390/pathogens10111453 (PMC8619305; doi:10.3390/pathogens10111453)
Supplement: Supplementary file 1 [file pathogens-10-01453-s001.zip › Supplementary materials, table T2.pdf]

# „Shining a LAMP" (Loop-Mediated Isothermal Amplification) on the molecular detection of Phytophthora spp. and Phytophthora cactorum in strawberry fields

Dominika G. Siegieda, Jacek Panek and Magdalena Frąc

**Table. T1.** Results of the detection of Phytophthora spp. and Phytophthora cactorum in environmental samples of organic strawberry fields. samples collected in 2019 and 2020.

| Sample | Plantation number | Coordinates | Type of the sample | Type of the soil | Strawberry variety | Time to positive *Phytophthora* spp. | Temperature of melting *Phytophthora* spp. | Time to positive *Phytophthora cactorum* | Temperature of melting *Phytophthora cactorum* |
|---|---|---|---|---|---|---|---|---|---|
| 385/19K | 4 | N49°55.8617' E022°31.0445' | roots | chernozem | Aprica | | | 23.28 | 91.09 |
| 449/19K | 10 | N50°08.4584' E023°00.1018' | roots | acrisol | Dipred | | | 32.91 | 90.13 |
| 45/19C | Mean from plantation 11 | - | bulk soil | acrisol | Aprica | | | 16.69 | 89.57 |
| 478/19 | 13 | N50°14.9756' E022°44.8757' | bulk soil | regosol | Aprica | | 90.15 | | |
| 490/19 | 14 | N50°14.9203' E022°44.8742' | bulk soil | nd | Aprica | 35.10 | 87.77 | | |
| 48/19C | Mean from plantations 1 and 2 | - | bulk soil | fluvisol | Aprica and Honeoye | 66.05 | 88.14 | | |
| 1468/20 | 15 | N51°35.7167' E021°64.5414' | fruit | nd | nd | | | 54.13 | 88.22 |
| 1469/20 | 15 | N51°35.7167' E021°64.5414' | fruit | nd | nd | | | 80.40 | 88.96 |
| 1472/20 | 15 | N51°35.7167' E021°64.5414' | fruit | nd | nd | | | 86.23 | 89.14 |
| 1485/20 | 15 | N51°35.7167' | fruit | nd | nd | | | 38.39 | 87.66 |

| | | | | | | | | | |
|---|---|---|---|---|---|---|---|---|---|
| | | E021°64.5414’ | | | | | | | |
| 1491/20 | 15 | N51°35.7167’ E021°64.5414’ | fruit | nd | nd | | | 72.46 | 89.33 |
| 1498/20 | 15 | N51°35.7167’ E021°64.5414’ | fruit | nd | nd | | | 80.96 | 88.96 |
| 1505/20 | 15 | N51°35.7167’ E021°64.5414’ | fruit | nd | nd | | | 78.07 | 88.96 |
| 1506/20 | 15 | N51°35.7167’ E021°64.5414’ | fruit | nd | nd | | | 84.41 | 88.96 |
| 1508/20 | 15 | N51°35.7167’ E021°64.5414’ | fruit | nd | nd | | | 81.19 | 89.14 |
| 1522/20 | 15 | N51°35.7167’ E021°64.5414’ | fruit | nd | nd | | | 81.88 | 88.96 |
| 1632/20 | 15 | N51°35.7167’ E021°64.5414’ | fruit | nd | Rumba | 66.67 | 90.32 | | |
| 347/19 | 1 | N50°07.8333' E022°37.5488' | rhizosphere | fluvisol | Aprica | | | | |
| 348/19 | 1 | N50°07.8263' E022°37.5166' | bulk soil | fluvisol | Aprica | | | | |
| 349/19 | 1 | N50°07.8212' E022°37.4898' | rhizosphere | fluvisol | Aprica | | | | |
| 350/19 | 1 | N50°07.8201' E022°37.4812' | bulk soil | fluvisol | Aprica | | | | |
| 351/19 | 1 | N50°07.8185' E022°37.4681' | rhizosphere | fluvisol | Aprica | | | | |
| 352/19K | 1 | N50°07.8333' E022°37.5488' | shoots | fluvisol | Aprica | | | | |
| 352/19L | 1 | N50°07.8333' E022°37.5488' | roots | fluvisol | Aprica | | | | |
| 354/19K | 1 | N50°07.8212' E022°37.4898' | shoots | fluvisol | Aprica | | | | |
| 354/19L | 1 | N50°07.8212' E022°37.4898' | roots | fluvisol | Aprica | | | | |
| 356/19L | 1 | N50°07.8185' E022°37.4681' | shoots | fluvisol | Aprica | | | | |

| | | | | | | | | | |
|---|---|---|---|---|---|---|---|---|---|
| 356/29K | 1 | N50°07.8185' E022°37.4681' | roots | fluvisol | Aprica | | | | |
| 337/19 | 2 | N50°07.7870' E022°37.4886' | bulk soil | fluvisol | Honeoye | | | | |
| 338/19 | 2 | N50°07.7906' E022°37.4944' | rhizosphere | fluvisol | Honeoye | | | | |
| 339/19 | 2 | N50°07.7941' E022°37.5015' | bulk soil | fluvisol | Honeoye | | | | |
| 340/19 | 2 | N50°07.7995' E022°37.5139' | rhizosphere | fluvisol | Honeoye | | | | |
| 341/19 | 2 | N50°07.8080' E022°37.5417' | bulk soil | fluvisol | Honeoye | | | | |
| 342/19K | 2 | N50°07.7870' E022°37.4886' | roots | fluvisol | Honeoye | | | | |
| 342/19L | 2 | N50°07.7870' E022°37.4886' | shoots | fluvisol | Honeoye | | | | |
| 343/19K | 2 | N50°07.7906' E022°37.4944' | roots | fluvisol | Honeoye | | | | |
| 343/19L | 2 | N50°07.7906' E022°37.4944' | shoots | fluvisol | Honeoye | | | | |
| 344/19K | 2 | N50°07.7941' E022°37.5015' | roots | fluvisol | Honeoye | | | | |
| 344/19L | 2 | N50°07.7941' E022°37.5015' | shoots | fluvisol | Honeoye | | | | |
| 357/19 | 3 | N50°07.3774' E022°36.8599' | rhizosphere | fluvisol | Aprica | | | | |
| 358/19 | 3 | N50°07.3881' E022°36.8722' | bulk soil | fluvisol | Dipred | | | | |
| 359/19 | 3 | N50°07.3955' E022°36.8877' | rhizosphere | fluvisol | Dipred | | | | |
| 360/19 | 3 | N50°07.4071' E022°36.9098' | bulk soil | fluvisol | Dipred | | | | |
| 361/19 | 3 | N50°07.4200' E022°36.9337' | rhizosphere | fluvisol | Dipred | | | | |
| 362/19K | 3 | N50°07.3774' E022°36.8599' | roots | fluvisol | Dipred | | | | |

| | | | | | | | | | |
|---|---|---|---|---|---|---|---|---|---|
| 362/19L | 3 | N50°07.3774' E022°36.8599' | shoots | fluvisol | Dipred | | | | |
| 364/19K | 3 | N50°07.3955' E022°36.8877' | roots | fluvisol | Dipred | | | | |
| 364/19L | 3 | N50°07.3955' E022°36.8877' | shoots | fluvisol | Dipred | | | | |
| 366/19K | 3 | N50°07.4200' E022°36.9337' | roots | fluvisol | Dipred | | | | |
| 366/19L | 3 | N50°07.4200' E022°36.9337' | shoots | fluvisol | Dipred | | | | |
| 53/19A | Mean from plantation 3 | - | bulk soil | fluvisol | Dipred | | | | |
| 53/19B | Mean from plantation 3 | - | bulk soil | fluvisol | Dipred | | | | |
| 53/19C | Mean from plantation 3 | - | bulk soil | fluvisol | Dipred | | | | |
| 379/19 | 4 | N49°55.8617' E022°31.0445' | rhizosphere | chernozem | Aprica | | | | |
| 380/19 | 4 | N49°55.8520' E022°31.0292' | bulk soil | chernozem | Aprica | | | | |
| 381/19 | 4 | N49°55.8339' E022°31.0084' | rhizosphere | chernozem | Aprica | | | | |
| 382/19 | 4 | N49°55.8150' E022°30.9833' | bulk soil | chernozem | Aprica | | | | |
| 383/19 | 4 | N49°55.7974' E022°30.9677' | rhizosphere | chernozem | Aprica | | | | |
| 384/19 | 4 | N49°55.7894' E022°30.9533' | bulk soil | chernozem | Aprica | | | | |
| 385/19L | 4 | N49°55.8617' E022°31.0445' | shoots | chernozem | Aprica | | | | |
| 387/19K | 4 | N49°55.8339' E022°31.0084' | roots | chernozem | Aprica | | | | |

| | | | | | | | | | |
|---|---|---|---|---|---|---|---|---|---|
| 387/19L | 4 | N49°55.8339' E022°31.0084' | shoots | chernozem | Aprica | | | | |
| 389/19K | 4 | N49°55.7974' E022°30.9677' | roots | chernozem | Aprica | | | | |
| 389/19L | 4 | N49°55.7974' E022°30.9677' | shoots | chernozem | Aprica | | | | |
| 538/19A | Mean from plantation 4 | - | bulk soil | acrisol | Aprica | | | | |
| 538/19B | Mean from plantation 4 | - | bulk soil | acrisol | Aprica | | | | |
| 538/19C | Mean from plantation 4 | - | bulk soil | acrisol | Aprica | | | | |
| 367/19 | 5 | N49°57.3395' E022°32.9881' | rhizosphere | cambisol | Honeoye | | | | |
| 368/19 | 5 | N49°57.3488' E022°32.9403' | bulk soil | cambisol | Honeoye | | | | |
| 369/19 | 5 | N49°57.3610' E022°32.8927' | rhizosphere | cambisol | Honeoye | | | | |
| 370/19 | 5 | N49°57.3695' E022°32.8507' | bulk soil | cambisol | Honeoye | | | | |
| 371/19 | 5 | N49°57.3825' E022°32.7908' | rhizosphere | cambisol | Honeoye | | | | |
| 372/19 | 5 | N49°57.3880' E022°32.7742' | bulk soil | cambisol | Honeoye | | | | |
| 373/19K | 5 | N49°57.3395' E022°32.9881' | roots | cambisol | Honeoye | | | | |
| 373/19L | 5 | N49°57.3395' E022°32.9881' | shoots | cambisol | Honeoye | | | | |
| 375/19K | 5 | N49°57.3610' E022°32.8927' | roots | cambisol | Honeoye | | | | |
| 375/19L | 5 | N49°57.3610' E022°32.8927' | shoots | cambisol | Honeoye | | | | |

| | | | | | | | | | |
|---|---|---|---|---|---|---|---|---|---|
| 377/19K | 5 | N49°57.3825' E022°32.7908' | roots | cambisol | Honeoye | | | | |
| 377/19L | 5 | N49°57.3825' E022°32.7908' | shoots | cambisol | Honeoye | | | | |
| 539/19A | Mean plantation 5 | - | bulk soil | acrisol | Honeoye | | | | |
| 539/19B | Mean plantation 5 | - | bulk soil | acrisol | Honeoye | | | | |
| 539/19C | Mean plantation 5 | - | bulk soil | acrisol | Honeoye | | | | |
| 391/19 | 6 | N49°58.8536' E022°39.4243' | rhizosphere | nd | Honeoye | | | | |
| 392/19 | 6 | N49°58.8490' E022°39.4429' | bulk soil | nd | Honeoye | | | | |
| 393/19 | 6 | N49°58.8406' E022°39.4630' | rhizosphere | nd | Honeoye | | | | |
| 394/19 | 6 | N49°58.8330' E022°39.4782' | bulk soil | nd | Honeoye | | | | |
| 395/19 | 6 | N49°58.8262' E022°39.4876' | rhizosphere | nd | Honeoye | | | | |
| 396/19 | 6 | N49°58.8160' E022°39.5011' | bulk soil | nd | Honeoye | | | | |
| 397/19K | 6 | N49°58.8536' E022°39.4243' | roots | nd | Honeoye | | | | |
| 397/19L | 6 | N49°58.8536' E022°39.4243' | shoots | nd | Honeoye | | | | |
| 399/19K | 6 | N49°58.8406' E022°39.4630' | roots | nd | Honeoye | | | | |
| 399/19L | 6 | N49°58.8406' E022°39.4630' | shoots | nd | Honeoye | | | | |
| 401/19K | 6 | N49°58.8262' E022°39.4876' | roots | nd | Honeoye | | | | |

| | | | | | | | | | |
|---|---|---|---|---|---|---|---|---|---|
| 401/19L | 6 | N49°58.8262' E022°39.4876' | shoots | nd | Honeoye | | | | |
| 415/19 | 7 | N49°50.9894' E022°48.1942' | rhizosphere | nd | Honeoye | | | | |
| 416/19 | 7 | N49°50.9811' E022°48.1870' | bulk soil | nd | Honeoye | | | | |
| 417/19 | 7 | N49°50.9689' E022°48.1791' | rhizosphere | nd | Honeoye | | | | |
| 418/19 | 7 | N49°50.9501' E022°48.1661' | bulk soil | nd | Honeoye | | | | |
| 419/19 | 7 | N49°50.9269' E022°48.1535' | rhizosphere | nd | Honeoye | | | | |
| 420/19 | 7 | N49°50.9030' E022°48.1427' | bulk soil | nd | Honeoye | | | | |
| 421/19K | 7 | N49°50.9894' E022°48.1942' | shoots | nd | Honeoye | | | | |
| 421/19L | 7 | N49°50.9894' E022°48.1942' | roots | nd | Honeoye | | | | |
| 423/19K | 7 | N49°50.9689' E022°48.1791' | shoots | nd | Honeoye | | | | |
| 423/19L | 7 | N49°50.9689' E022°48.1791' | roots | nd | Honeoye | | | | |
| 425/19L | 7 | N49°50.9269' E022°48.1535' | shoots | nd | Honeoye | | | | |
| 426/19K | 7 | N49°50.9269' E022°48.1535' | roots | nd | Honeoye | | | | |
| 427/19 | 8 | N49°50.9375' E022°48.1501' | rhizosphere | nd | Honeoye | | | | |
| 428/19 | 8 | N49°50.9312' E022°48.1438' | bulk soil | nd | Honeoye | | | | |
| 429/19 | 8 | N49°50.9237' E022°48.1383' | rhizosphere | nd | Honeoye | | | | |
| 430/19 | 8 | N49°50.9227' E022°48.1366' | bulk soil | nd | Honeoye | | | | |
| 431/19 | 8 | N49°50.9132' E022°48.1280' | rhizosphere | nd | Honeoye | | | | |

| | | | | | | | | | |
|---|---|---|---|---|---|---|---|---|---|
| 432/19 | 8 | N49°50.9069' E022°48.1229' | bulk soil | nd | Honeoye | | | | |
| 433/19K | 8 | N49°50.9375' E022°48.1501' | roots | nd | Honeoye | | | | |
| 433/19L | 8 | N49°50.9375' E022°48.1501' | shoots | nd | Honeoye | | | | |
| 435/19K | 8 | N49°50.9237' E022°48.1383' | roots | nd | Honeoye | | | | |
| 435/19L | 8 | N49°50.9237' E022°48.1383' | shoots | nd | Honeoye | | | | |
| 437/19K | 8 | N49°50.9132' E022°48.1280' | roots | nd | Honeoye | | | | |
| 437/19L | 8 | N49°50.9132' E022°48.1280' | shoots | nd | Honeoye | | | | |
| 403/19 | 9 | N49°50.9225' E022°48.1597' | bulk soil | nd | Honeoye | | | | |
| 404/19 | 9 | N49°50.9343' E022°48.1676' | rhizosphere | nd | Honeoye | | | | |
| 405/19 | 9 | N49°50.9454' E022°48.1765' | bulk soil | nd | Honeoye | | | | |
| 406/19 | 9 | N49°50.9599' E022°48.1877' | rhizosphere | nd | Honeoye | | | | |
| 407/19 | 9 | N49°50.9723' E022°48.1978' | bulk soil | nd | Honeoye | | | | |
| 408/19 | 9 | N49°50.9809' E022°48.2039' | rhizosphere | nd | Honeoye | | | | |
| 410/19K | 9 | N49°50.9343' E022°48.1676' | roots | nd | Honeoye | | | | |
| 410/19L | 9 | N49°50.9343' E022°48.1676' | shoots | nd | Honeoye | | | | |
| 412/19K | 9 | N49°50.9599' E022°48.1877' | roots | nd | Honeoye | | | | |
| 412/19L | 9 | N49°50.9599' E022°48.1877' | shoots | nd | Honeoye | | | | |
| 414/19K | 9 | N49°50.9809' E022°48.2039' | roots | nd | Honeoye | | | | |

| | | | | | | | | | |
|---|---|---|---|---|---|---|---|---|---|
| 414/19L | 9 | N49°50.9809'<br>E022°48.2039' | shoots | nd | Honeoye | | | | |
| 439/19 | 10 | N50°08.5010'<br>E023°00.1346' | rhizosphere | acrisol | Dipred | | | | |
| 440/19 | 10 | N50°08.4844'<br>E023°00.1220' | bulk soil | acrisol | Dipred | | | | |
| 441/19 | 10 | N50°08.4775'<br>E023°00.1167' | rhizosphere | acrisol | Dipred | | | | |
| 442/19 | 10 | N50°08.4652'<br>E023°00.1084' | bulk soil | acrisol | Dipred | | | | |
| 443/19 | 10 | N50°08.4584'<br>E023°00.1018' | rhizosphere | acrisol | Dipred | | | | |
| 444/19 | 10 | N50°08.4585'<br>E023°00.0963' | bulk soil | acrisol | Dipred | | | | |
| 445/19K | 10 | N50°08.5010'<br>E023°00.1346' | roots | acrisol | Dipred | | | | |
| 445/19L | 10 | N50°08.5010'<br>E023°00.1346' | shoots | acrisol | Dipred | | | | |
| 447/19K | 10 | N50°08.4775'<br>E023°00.1167' | roots | acrisol | Dipred | | | | |
| 447/19L | 10 | N50°08.4775'<br>E023°00.1167' | shoots | acrisol | Dipred | | | | |
| 449/19L | 10 | N50°08.4584'<br>E023°00.1018' | shoots | acrisol | Dipred | | | | |
| 45/19A | Mean from plantation 11 | - | bulk soil | acrisol | Aprica | | | | |
| 45/19B | Mean from plantation 11 | - | bulk soil | acrisol | Aprica | | | | |
| 451/19 | 11 | N50°08.4642'<br>E023°00.0965' | rhizosphere | acrisol | Dipred | | | | |
| 452/19 | 11 | N50°08.4802'<br>E023°00.1045' | bulk soil | acrisol | Dipred | | | | |

| | | | | | | | | | |
|---|---|---|---|---|---|---|---|---|---|
| 453/19 | 11 | N50°08.4880' E023°00.1081' | rhizosphere | acrisol | Dipred | | | | |
| 454/19 | 11 | 50.141638, 23.001876 | bulk soil | acrisol | Dipred | | | | |
| 455/19 | 11 | 50.141820 23.001952 | rhizosphere | acrisol | Dipred | | | | |
| 456/19 | 11 | N50°08.5164' E023°00.1201' | bulk soil | acrisol | Dipred | | | | |
| 457/19K | 11 | N50°08.4642' E023°00.0965' | roots | acrisol | Dipred | | | | |
| 457/19L | 11 | N50°08.4642' E023°00.0965' | shoots | acrisol | Dipred | | | | |
| 459/19K | 11 | N50°08.4880' E023°00.1081' | roots | acrisol | Dipred | | | | |
| 459/19L | 11 | N50°08.4880' E023°00.1081' | shoots | acrisol | Dipred | | | | |
| 461/19K | 11 | 50.141820 23.001952 | roots | acrisol | Aprica | | | | |
| 461/19L | 11 | 50.141820 23.001952 | shoots | acrisol | Aprica | | | | |
| 57/19A | Mean from plantation 11 | - | bulk soil | acrisol | Dipred | | | | |
| 57/19B | Mean from plantation 11 | - | bulk soil | acrisol | Dipred | | | | |
| 57/19C | Mean from plantation 11 | - | bulk soil | acrisol | Dipred | | | | |
| 43/19A | Mean from plantation 12 | - | bulk soil | acrisol | Honeoye | | | | |
| 43/19B | Mean from plantation 12 | - | bulk soil | acrisol | Honeoye | | | | |

| | | | | | | | | | |
|---|---|---|---|---|---|---|---|---|---|
| 43/19C | Mean from plantation 12 | - | bulk soil | acrisol | Honeoye | | | | |
| 463/19 | 12 | N50°08.9259' E023°00.1276' | rhizosphere | acrisol | Honeoye | | | | |
| 464/19 | 12 | N50°08.9400' E023°00.1382' | bulk soil | acrisol | Honeoye | | | | |
| 465/19 | 12 | N50°08.9551' E023°00.1541' | rhizosphere | acrisol | Honeoye | | | | |
| 466/19 | 12 | N50°08.9668' E023°00.1659' | bulk soil | acrisol | Honeoye | | | | |
| 467/19 | 12 | N50°08.9887' E023°00.1803' | rhizosphere | acrisol | Honeoye | | | | |
| 468/19 | 12 | N50°09.0160' E023°00.1975' | bulk soil | acrisol | Honeoye | | | | |
| 469/19K | 12 | N50°08.9259' E023°00.1276' | roots | acrisol | Honeoye | | | | |
| 469/19L | 12 | N50°08.9259' E023°00.1276' | shoots | acrisol | Honeoye | | | | |
| 471/19K | 12 | N50°08.9551' E023°00.1541' | roots | acrisol | Honeoye | | | | |
| 471/19L | 12 | N50°08.9551' E023°00.1541' | shoots | acrisol | Honeoye | | | | |
| 473/19K | 12 | N50°08.9887' E023°00.1803' | roots | acrisol | Honeoye | | | | |
| 473/19L | 12 | N50°08.9887' E023°00.1803' | shoots | acrisol | Honeoye | | | | |
| 475/19 | 13 | N50°14.9959' E022°44.8649' | rhizosphere | regosol | Aprica | | | | |
| 476/19 | 13 | N50°14.9931' E022°44.8690' | bulk soil | regosol | Aprica | | | | |
| 477/19 | 13 | N50°14.9841' E022°44.8706' | rhizosphere | regosol | Aprica | | | | |
| 479/19 | 13 | N50°14.9756' E022°44.8757' | rhizosphere | regosol | Aprica | | | | |

| | | | | | | | | | |
|---|---|---|---|---|---|---|---|---|---|
| 480/19 | 13 | N50°14.9683' E022°44.8815' | bulk soil | regosol | Aprica | | | | |
| 481/19K | 13 | N50°14.9959' E022°44.8649' | roots | regosol | Aprica | | | | |
| 481/19L | 13 | N50°14.9959' E022°44.8649’ | shoots | regosol | Aprica | | | | |
| 483/19K | 13 | N50°14.9841' E022°44.8706' | roots | regosol | Aprica | | | | |
| 483/19L | 13 | N50°14.9841' E022°44.8706' | shoots | regosol | Aprica | | | | |
| 485/19K | 13 | N50°14.9722' E022°44.8806' | roots | regosol | Aprica | | | | |
| 485/19L | 13 | N50°14.9722' E022°44.8806' | shoots | regosol | Aprica | | | | |
| 487/19 | 14 | N50°14.9445' E022°44.8819' | rhizosphere | | Aprica | | | | |
| 488/19 | 14 | 50.248953, 22.747947 | bulk soil | | Aprica | | | | |
| 489/19 | 14 | N50°14.9281' E022°44.8763' | rhizosphere | | Aprica | | | | |
| 491/19 | 14 | N50°14.9133' E022°44.8697' | rhizosphere | | Aprica | | | | |
| 492/19 | 14 | N50°14.9025' E022°44.8702' | bulk soil | | Aprica | | | | |
| 493/19K | 14 | N50°14.9445' E022°44.8819' | roots | | Aprica | | | | |
| 493/19L | 14 | N50°14.9445' E022°44.8819' | shoots | | Aprica | | | | |
| 493/19L | 14 | N50°14.9281' E022°44.8763' | shoots | | Aprica | | | | |
| 495/19K | 14 | N50°14.9281' E022°44.8763' | roots | | Aprica | | | | |
| 495/19L | 14 | N50°14.9133' E022°44.8697' | shoots | | Aprica | | | | |
| 497/19K | 14 | N50°14.9133' E022°44.8697' | roots | | Aprica | | | | |

| | | | | | | | | | |
|---|---|---|---|---|---|---|---|---|---|
| 48/19A | Mean from plantations 1 and 2 | - | bulk soil | fluvisol | Aprica and Honeoye | | | | |
| 48/19B | Mean from plantations 1 and 2 | - | bulk soil | fluvisol | Aprica and Honeoye | | | | |
| 32/19A | Mean from plantations 13 and 14 | - | bulk soil | regosol | Aprica | | | | |
| 55/19A | Mean from plantations 13 and 14 | - | bulk soil | regosol | Aprica | | | | |
| 55/19B | Mean from plantations 13 and 14 | - | bulk soil | regosol | Aprica | | | | |
| 55/19C | Mean from plantations 13 and 14 | - | bulk soil | regosol | Aprica | | | | |
| 32/19B | Mean from plantations 13 and 14 | - | bulk soil | regosol | Aprica | | | | |
| 32/19C | Mean from plantations 13 and 14 | - | bulk soil | regosol | Aprica | | | | |
| 1470/20 | 15 | N51°35.7167’ E021°64.5414’ | fruit | nd | nd | | | | |
| 1471/20 | 15 | N51°35.7167’ E021°64.5414’ | fruit | nd | nd | | | | |
| 1473/20 | 15 | N51°35.7167’ E021°64.5414’ | fruit | nd | nd | | | | |
| 1474/20 | 15 | N51°35.7167’ E021°64.5414’ | fruit | nd | nd | | | | |
| 1475/20 | 15 | N51°35.7167’ E021°64.5414’ | fruit | nd | nd | | | | |

| | | | | | | | | | |
|---|---|---|---|---|---|---|---|---|---|
| 1476/20 | 15 | N51°35.7167’ E021°64.5414’ | fruit | nd | nd | | | | |
| 1477/20 | 15 | N51°35.7167’ E021°64.5414’ | fruit | nd | nd | | | | |
| 1478/20 | 15 | N51°35.7167’ E021°64.5414’ | fruit | nd | nd | | | | |
| 1479/20 | 15 | N51°35.7167’ E021°64.5414’ | fruit | nd | nd | | | | |
| 1480/20 | 15 | N51°35.7167’ E021°64.5414’ | fruit | nd | nd | | | | |
| 1481/20 | 15 | N51°35.7167’ E021°64.5414’ | fruit | nd | nd | | | | |
| 1482/20 | 15 | N51°35.7167’ E021°64.5414’ | fruit | nd | nd | | | | |
| 1483/20 | 15 | N51°35.7167’ E021°64.5414’ | fruit | nd | nd | | | | |
| 1484/20 | 15 | N51°35.7167’ E021°64.5414’ | fruit | nd | nd | | | | |
| 1486/20 | 15 | N51°35.7167’ E021°64.5414’ | fruit | nd | nd | | | | |
| 1487/20 | 15 | N51°35.7167’ E021°64.5414’ | fruit | nd | nd | | | | |
| 1488/20 | 15 | N51°35.7167’ E021°64.5414’ | fruit | nd | nd | | | | |
| 1489/20 | 15 | N51°35.7167’ E021°64.5414’ | fruit | nd | nd | | | | |
| 1490/20 | 15 | N51°35.7167’ E021°64.5414’ | fruit | nd | nd | | | | |
| 1492/20 | 15 | N51°35.7167’ E021°64.5414’ | fruit | nd | nd | | | | |
| 1493/20 | 15 | N51°35.7167’ E021°64.5414’ | fruit | nd | nd | | | | |
| 1494/20 | 15 | N51°35.7167’ E021°64.5414’ | fruit | nd | nd | | | | |
| 1495/20 | 15 | N51°35.7167’ E021°64.5414’ | fruit | nd | nd | | | | |

| | | | | | | | | | |
|---|---|---|---|---|---|---|---|---|---|
| 1496/20 | 15 | N51°35.7167’ E021°64.5414’ | fruit | nd | nd | | | | |
| 1497/20 | 15 | N51°35.7167’ E021°64.5414’ | fruit | nd | nd | | | | |
| 1499/20 | 15 | N51°35.7167’ E021°64.5414’ | fruit | nd | nd | | | | |
| 1500/20 | 15 | N51°35.7167’ E021°64.5414’ | fruit | nd | nd | | | | |
| 1501/20 | 15 | N51°35.7167’ E021°64.5414’ | fruit | nd | nd | | | | |
| 1502/20 | 15 | N51°35.7167’ E021°64.5414’ | fruit | nd | nd | | | | |
| 1503/20 | 15 | N51°35.7167’ E021°64.5414’ | fruit | nd | nd | | | | |
| 1504/20 | 15 | N51°35.7167’ E021°64.5414’ | fruit | nd | nd | | | | |
| 1507/20 | 15 | N51°35.7167’ E021°64.5414’ | fruit | nd | nd | | | | |
| 1509/20 | 15 | N51°35.7167’ E021°64.5414’ | fruit | nd | nd | | | | |
| 1510/20 | 15 | N51°35.7167’ E021°64.5414’ | fruit | nd | nd | | | | |
| 1511/20 | 15 | N51°35.7167’ E021°64.5414’ | fruit | nd | nd | | | | |
| 1512/20 | 15 | N51°35.7167’ E021°64.5414’ | fruit | nd | nd | | | | |
| 1513/20 | 15 | N51°35.7167’ E021°64.5414’ | fruit | nd | nd | | | | |
| 1514/20 | 15 | N51°35.7167’ E021°64.5414’ | fruit | nd | nd | | | | |
| 1515/20 | 15 | N51°35.7167’ E021°64.5414’ | fruit | nd | nd | | | | |
| 1516/20 | 15 | N51°35.7167’ E021°64.5414’ | fruit | nd | nd | | | | |
| 1517/20 | 15 | N51°35.7167’ E021°64.5414’ | fruit | nd | nd | | | | |

| | | | | | | | | | |
|---|---|---|---|---|---|---|---|---|---|
| 1518/20 | 15 | N51°35.7167’ E021°64.5414’ | fruit | nd | nd | | | | |
| 1519/20 | 15 | N51°35.7167’ E021°64.5414’ | fruit | nd | nd | | | | |
| 1520/20 | 15 | N51°35.7167’ E021°64.5414’ | fruit | nd | nd | | | | |
| 1521/20 | 15 | N51°35.7167’ E021°64.5414’ | fruit | nd | nd | | | | |
| 1523/20 | 15 | N51°35.7167’ E021°64.5414’ | fruit | nd | nd | | | | |
| 1524/20 | 15 | N51°35.7167’ E021°64.5414’ | fruit | nd | nd | | | | |
| 1525/20 | 15 | N51°35.7167’ E021°64.5414’ | fruit | nd | nd | | | | |
| 1526/20 | 15 | N51°35.7167’ E021°64.5414’ | fruit | nd | nd | | | | |
| 1527/20 | 15 | N51°35.7167’ E021°64.5414’ | fruit | nd | nd | | | | |
| 1528/20 | 15 | N51°35.7167’ E021°64.5414’ | fruit | nd | nd | | | | |
| 1529/20 | 15 | N51°35.7167’ E021°64.5414’ | fruit | nd | nd | | | | |
| 1530/20 | 15 | N51°35.7167’ E021°64.5414’ | fruit | nd | nd | | | | |
| 1531/20 | 15 | N51°35.7167’ E021°64.5414’ | fruit | nd | nd | | | | |
| 1532/20 | 15 | N51°35.7167’ E021°64.5414’ | fruit | nd | nd | | | | |
| 1533/20 | 15 | N51°35.7167’ E021°64.5414’ | fruit | nd | nd | | | | |
| 1534/20 | 15 | N51°35.7167’ E021°64.5414’ | fruit | nd | nd | | | | |
| 1535/20 | 15 | N51°35.7167’ E021°64.5414’ | fruit | nd | nd | | | | |
| 1536/20 | 15 | N51°35.7167’ E021°64.5414’ | fruit | nd | nd | | | | |

| | | | | | | | | | |
|---|---|---|---|---|---|---|---|---|---|
| 1537/20 | 15 | N51°35.7167’ E021°64.5414’ | fruit | nd | nd | | | | |
| 1538/20 | 15 | N51°35.7167’ E021°64.5414’ | fruit | nd | nd | | | | |
| 1539/20 | 15 | N51°35.7167’ E021°64.5414’ | fruit | nd | nd | | | | |
| 1540/20 | 15 | N51°35.7167’ E021°64.5414’ | fruit | nd | nd | | | | |
| 1541/20 | 15 | N51°35.7167’ E021°64.5414’ | fruit | nd | nd | | | | |
| 1542/20 | 15 | N51°35.7167’ E021°64.5414’ | fruit | nd | nd | | | | |
| 1543/20 | 15 | N51°35.7167’ E021°64.5414’ | fruit | nd | nd | | | | |
| 1544/20 | 15 | N51°35.7167’ E021°64.5414’ | fruit | nd | nd | | | | |
| 1545/20 | 15 | N51°35.7167’ E021°64.5414’ | fruit | nd | nd | | | | |
| 1546/20 | 15 | N51°35.7167’ E021°64.5414’ | fruit | nd | nd | | | | |
| 1547/20 | 15 | N51°35.7167’ E021°64.5414’ | fruit | nd | nd | | | | |
| 1548/20 | 15 | N51°35.7167’ E021°64.5414’ | fruit | nd | nd | | | | |
| 1549/20 | 15 | N51°35.7167’ E021°64.5414’ | fruit | nd | nd | | | | |
| 1550/20 | 15 | N51°35.7167’ E021°64.5414’ | fruit | nd | nd | | | | |
| 1551/20 | 15 | N51°35.7167’ E021°64.5414’ | fruit | nd | nd | | | | |
| 1552/20 | 15 | N51°35.7167’ E021°64.5414’ | fruit | nd | nd | | | | |
| 1553/20 | 15 | N51°35.7167’ E021°64.5414’ | fruit | nd | nd | | | | |
| 1554/20 | 15 | N51°35.7167’ E021°64.5414’ | fruit | nd | nd | | | | |

| | | | | | | | | | |
|---|---|---|---|---|---|---|---|---|---|
| 1555/20 | 15 | N51°35.7167' E021°64.5414' | fruit | nd | nd | | | | |
| 1556/20 | 15 | N51°35.7167' E021°64.5414' | fruit | nd | nd | | | | |
| 1557/20 | 15 | N51°35.7167' E021°64.5414' | fruit | nd | nd | | | | |
| 1558/20 | 15 | N51°35.7167' E021°64.5414' | fruit | nd | nd | | | | |
| 1559/20 | 15 | N51°35.7167' E021°64.5414' | fruit | nd | nd | | | | |
| 1560/20 | 15 | N51°35.7167' E021°64.5414' | fruit | nd | nd | | | | |
| 1561/20 | 15 | N51°35.7167' E021°64.5414' | fruit | nd | nd | | | | |
| 1562/20 | 15 | N51°35.7167' E021°64.5414' | fruit | nd | nd | | | | |
| 1563/20 | 15 | N51°35.7167' E021°64.5414' | fruit | nd | nd | | | | |
| 1564/20 | 15 | N51°35.7167' E021°64.5414' | fruit | nd | nd | | | | |
| 1565/20 | 15 | N51°35.7167' E021°64.5414' | fruit | nd | nd | | | | |
| 1566/20 | 15 | N51°35.7167' E021°64.5414' | fruit | nd | nd | | | | |
| 1567/20 | 15 | N51°35.7167' E021°64.5414' | fruit | nd | nd | | | | |
| 1568/20 | 15 | N51°35.7167' E021°64.5414' | fruit | nd | nd | | | | |
| 1569/20 | 15 | N51°35.7167' E021°64.5414' | fruit | nd | nd | | | | |
| 1570/20 | 15 | N51°35.7167' E021°64.5414' | fruit | nd | nd | | | | |
| 1571/20 | 15 | N51°35.7167' E021°64.5414' | fruit | nd | nd | | | | |
| 1572/20 | 15 | N51°35.7167' E021°64.5414' | fruit | nd | nd | | | | |

| | | | | | | | | | |
|---|---|---|---|---|---|---|---|---|---|
| 1573/20 | 15 | N51°35.7167’ E021°64.5414’ | fruit | nd | nd | | | | |
| 1574/20 | 15 | N51°35.7167’ E021°64.5414’ | fruit | nd | nd | | | | |
| 1575/20 | 15 | N51°35.7167’ E021°64.5414’ | fruit | nd | nd | | | | |
| 1576/20 | 15 | N51°35.7167’ E021°64.5414’ | fruit | nd | nd | | | | |
| 1577/20 | 15 | N51°35.7167’ E021°64.5414’ | fruit | nd | nd | | | | |
| 1578/20 | 15 | N51°35.7167’ E021°64.5414’ | fruit | nd | nd | | | | |
| 1579/20 | 15 | N51°35.7167’ E021°64.5414’ | fruit | nd | nd | | | | |
| 1580/20 | 15 | N51°35.7167’ E021°64.5414’ | fruit | nd | nd | | | | |
| 1581/20 | 15 | N51°35.7167’ E021°64.5414’ | fruit | nd | nd | | | | |
| 1624/20 | 15 | N51°35.7167’ E021°64.5414’ | fruit | nd | Honeoye | | | | |
| 1625/20 | 15 | N51°35.7167’ E021°64.5414’ | fruit | nd | Vibrant | | | | |
| 1626/20 | 15 | N51°35.7167’ E021°64.5414’ | fruit | nd | Rumba | | | | |
| 1627/20 | 15 | N51°35.7167’ E021°64.5414’ | fruit | nd | Honeoye | | | | |
| 1628/20 | 15 | N51°35.7167’ E021°64.5414’ | fruit | nd | Vibrant | | | | |
| 1629/20 | 15 | N51°35.7167’ E021°64.5414’ | fruit | nd | Rumba | | | | |
| 1630/20 | 15 | N51°35.7167’ E021°64.5414’ | fruit | nd | Honeoye | | | | |
| 1631/20 | 15 | N51°35.7167’ E021°64.5414’ | fruit | nd | Vibrant | | | | |
| 1633/20 | 15 | N51°35.7167’ E021°64.5414’ | fruit | nd | Honeoye | | | | |

| | | | | | | | | | |
|---|---|---|---|---|---|---|---|---|---|
| 1634/20 | 15 | N51°35.7167’ E021°64.5414’ | fruit | nd | Vibrant | | | | |
| 1635/20 | 15 | N51°35.7167’ E021°64.5414’ | fruit | nd | Rumba | | | | |
| 1636/20 | 15 | N51°35.7167’ E021°64.5414’ | fruit | nd | Honeoye | | | | |
| 1637/20 | 15 | N51°35.7167’ E021°64.5414’ | fruit | nd | Vibrant | | | | |
| 1638/20 | 15 | N51°35.7167’ E021°64.5414’ | fruit | nd | Rumba | | | | |
| 1639/20 | 15 | N51°35.7167’ E021°64.5414’ | fruit | nd | Honeoye | | | | |
| 1640/20 | 15 | N51°35.7167’ E021°64.5414’ | fruit | nd | Vibrant | | | | |
| 1641/20 | 15 | N51°35.7167’ E021°64.5414’ | fruit | nd | Rumba | | | | |
| 1642/20 | 15 | N51°35.7167’ E021°64.5414’ | fruit | nd | Honeoye | | | | |
| 1643/20 | 15 | N51°35.7167’ E021°64.5414’ | fruit | nd | Vibrant | | | | |
| 1644/20 | 15 | N51°35.7167’ E021°64.5414’ | fruit | nd | Rumba | | | | |
| 1645/20 | 15 | N51°35.7167’ E021°64.5414’ | fruit | nd | Honeoye | | | | |
| 1646/20 | 15 | N51°35.7167’ E021°64.5414’ | fruit | nd | Vibrant | | | | |
| 1647/20 | 15 | N51°35.7167’ E021°64.5414’ | fruit | nd | Rumba | | | | |
| 1648/20 | 15 | N51°35.7167’ E021°64.5414’ | fruit | nd | Rumba | | | | |
| 1649/20 | 15 | N51°35.7167’ E021°64.5414’ | fruit | nd | Honeoye | | | | |
| 1650/20 | 15 | N51°35.7167’ E021°64.5414’ | fruit | nd | Vibrant | | | | |
| 1651/20 | 15 | N51°35.7167’ E021°64.5414’ | fruit | nd | Honeoye | | | | |

| | | | | | | | | | |
|---|---|---|---|---|---|---|---|---|---|
| 1652/20 | 15 | N51°35.7167’ E021°64.5414’ | fruit | nd | Vibrant | | | | |
| 1653/20 | 15 | N51°35.7167’ E021°64.5414’ | fruit | nd | Rumba | | | | |
| 1654/20 | 15 | N51°35.7167’ E021°64.5414’ | fruit | nd | Honeoye | | | | |
| 1655/20 | 15 | N51°35.7167’ E021°64.5414’ | fruit | nd | Vibrant | | | | |
| 1656/20 | 15 | N51°35.7167’ E021°64.5414’ | fruit | nd | Rumba | | | | |
| 1657/20 | 15 | N51°35.7167’ E021°64.5414’ | fruit | nd | Honeoye | | | | |
| 1658/20 | 15 | N51°35.7167’ E021°64.5414’ | fruit | nd | Vibrant | | | | |
| 1659/20 | 15 | N51°35.7167’ E021°64.5414’ | fruit | nd | Rumba | | | | |
| 1660/20 | 15 | N51°35.7167’ E021°64.5414’ | fruit | nd | Honeoye | | | | |
| 1661/20 | 15 | N51°35.7167’ E021°64.5414’ | fruit | nd | Vibrant | | | | |
| 1662/20 | 15 | N51°35.7167’ E021°64.5414’ | fruit | nd | Rumba | | | | |
| 1663/20 | 15 | N51°35.7167’ E021°64.5414’ | fruit | nd | Honeoye | | | | |
| 1664/20 | 15 | N51°35.7167’ E021°64.5414’ | fruit | nd | Vibrant | | | | |
| 1665/20 | 15 | N51°35.7167’ E021°64.5414’ | fruit | nd | Rumba | | | | |
| | | | | | | | | | |
| | 2019 | | 192 | | | 4 | 4 | 13 | 13 |
| | 2020 | | 156 | | | | | | |
| | SUM | | 331 | | | percentage | 1% | 4% | |
| | | | | | | | | | |
| | | | | | | | | | |

| | | | | | | Time to positive *Phytophthora* spp. | Temperature of melting *Phytophthora* spp. | Time to positive *Phytophthora cactorum* | Temperature of melting *Phytophthora cactorum* |
|---|---|---|---|---|---|---|---|---|---|
| | | | | | max | 66.67 | 90.32 | 86.23 | 91.09 |
| | | | | | min | 35.10 | 87.77 | 16.69 | 87.66 |
